# A Comprehensive Gene Expression Profile of Pectin Degradation Enzymes Reveals the Molecular Events during Cell Wall Degradation and Pathogenesis of Rice Sheath Blight Pathogen *Rhizoctonia solani* AG1-IA

**DOI:** 10.3390/jof6020071

**Published:** 2020-05-25

**Authors:** Talluri Bhaskar Rao, Ramakrishna Chopperla, Naresh Babu Prathi, Marudamuthu Balakrishnan, Vellaisamy Prakasam, Gouri Sankar Laha, Sena Munuswamy Balachandran, Satendra K. Mangrauthia

**Affiliations:** 1 Indian Council of Agricultural Research-Indian Institute of Rice Research, Hyderabad 500030, India; talluribhaskar31@gmail.com (T.B.R.); chopperlaramakrishna@gmail.com (R.C.); nareshseshu0962@gmail.com (N.B.P.); vprakasam.iari@gmail.com (V.P.); lahags@yahoo.co.in (G.S.L.); balasena@yahoo.com (S.M.B.); 2 Bioinformatics Lab, ICAR- National Academy of Agricultural Research Management, Hyderabad 500030, India; balakrishnan@naarm.ernet.in

**Keywords:** transcriptome, strains, polygalacturonase, qRT-PCR, *Oryza sativa*, cell wall degrading enzymes

## Abstract

Sheath blight disease of rice caused by *Rhizoctonia solani* Kühn (teleomorph: *Thanatephorus cucumeris*) remains a global challenge due to the absence of reliable resistance genes and poor understanding of pathogen biology. Pectin, one of the most vital constituents of the plant cell wall, is targeted by pectin methylesterases, polygalacturonases, and few other enzymes of fungal pathogens. In this study, we catalogued the expressed genes of the fungal genome from RNAseq of *R. solani* infected four rice genotypes. Analysis of RNAseq revealed 3325 pathogen genes commonly expressed in all rice genotypes, in which 49, 490, and 83 genes were specific to BPT5204, Tetep, and Pankaj genotypes, respectively. To identify the early and late responding genes of *R. solani* during plant cell wall degradation, a real-time PCR analysis of 30 pectinolytic enzymes was done at six different time points after inoculation. The majority of these genes showed maximum induction at the 72 h time point, suggesting that it is the most crucial stage of infection. Pankaj showed lesser induction of these genes as compared to other genotypes. Leaf-blade tissue and 45 days old-growth stage are more favorable for the expression of pectin degradation genes of *R. solani*. Additionally, the expression analysis of these genes from four different strains of *R. solani* suggested differential regulation of genes but no distinct expression pattern between highly virulent and mild strains. The implications of the differential regulation of these genes in disease development have been discussed. This study provides the first such comprehensive analysis of *R. solani* genes encoding pectin degrading enzymes, which would help to decipher the pathogen biology and sheath blight disease development.

## 1. Introduction

*Rhizoctonia solani* Kühn (teleomorph: *Thanatephorus cucumeris* (A. B. Frank) Donk.) is a common soil-borne Basidiomycete fungus that can infect a vast range of plant species including field crops, horticulture crops, and weeds [1,2]. The pathogen is classified into 14 anastomosis groups (AG1 to AG13, and AGBI) based on distinct physiology, genetic composition, and hyphal interactions. The AG1 IA, an intra-specific member of *R. solani* AG1 group, is considered as the most devastating pathogen causing aerial blight, brown patch, banded leaf, and sheath blight diseases [3,4]. The sheath blight caused by this necrotrophic fungus is one of the most destructive diseases of rice that affects the grain quality and can cause yield loss up to 50% under a favorable environment [5,6]. The unavailability of desirable genetic resistance against *R. solani* AG1 IA in commercial rice cultivars prompted researchers to make serious attempts to develop sheath blight disease-resistant cultivars through breeding and transgenic approaches [7,8,9,10]. However, translation of these efforts into the development of highly resistant commercial cultivars could not be realized due to several factors, and one among them is the lack of a clear understanding of molecular mechanisms of disease initiation and establishment. Resistance breeding is more successful in cases where pathogen biology is very well understood [11,12,13]. In recent years, efforts to understand the pathogen biology have been intensified through genomics, transcriptomics, and proteomics approaches [4,14,15,16,17]. Such studies also help in developing the more effective chemical molecules for controlling the pathogen through the use of fungicides. 

RNA-sequencing (RNAseq) is the most widely used tool to understand the functional elements of host and pathogen genomes during the host–pathogen interaction and disease development. It utilizes the deep sequencing technologies for precise cataloging of the transcriptome in a given cell or tissue [18]. RNAseq has been used to reveal the transcriptome of *Magnaporthe oryzae* during rice blast disease [19], *Sclerotinia sclerotiorum* during white mold disease of brassica [20], *Ustilago maydis* during maize smut disease [21], and several other pathosystems [22]. It has also been used to unravel the transcriptomics of *R. solani* AG1-IB during the bottom-rot of lettuce [23], *R. solani* AG1-IA during foliar blight of soybean, and brown spot of *Zoysia japonica* [24,25]. RNAseq has also been used to understand the transcriptomic dynamics of sclerotial development [17], candidate pathogenicity determinants [15], and host selection fitness mechanisms [26] of *R. solani* AG1-IA causing rice sheath bight disease.

Plant cell wall degradation by the fungal pathogens is necessary for penetration of host tissue and branching of hyphae inside the cell. This helps in releasing the necessary nutrients for the growth and multiplication of fungal pathogens, thus helping in not only the infection process but also disease establishment [27,28]. The activity of cell wall degrading enzymes (CWDEs) determines the degree of pathogenesis [29,30]. Among the CWDEs, pectin degrading enzymes are considered as most crucial for fungal infection of plants by allowing the cellulases and hemicellulases to act on exposed cell wall components for further degradation [31]. The pectin matrix of plant cell wall is the primary target of fungal pathogens; hence, plants alter methylesterification status of pectin during infection [32,33]. Consequently, fungal pathogens have also evolved several pectin degrading enzymes as a counter-defense strategy. Pectin methylesterases (PME) and polygalacturonase (PG) are key enzymes associated with de-methylation of pectin to pectate and hydrolysis of pectate by cleaving the α-(1,4)-glycosidic bonds, respectively. The activity of these enzymes of *R. solani* AG1-IA was studied during rice sheath blight and peanut sheath blight diseases [28,34], and a strong association between enzyme activity and fungal pathogenicity was established. Pectin lyases produce unsaturated methyl-oligo-galacturonates by catalyzing the random cleavage of highly esterified pectin [35]. These CWDEs are encoded by several genes; therefore, enzyme activity assays do not distinguish the role of individual genes in infection and disease development process. Further, efforts have not been made to develop the comprehensive profile of the expression pattern of genes encoding pectin degradation enzymes during rice sheath blight disease. In addition to plant cell wall degradation, cytochrome P450 mediated cellular defense and gene regulation by transcription factors are essential biological processes of fungi, which help in rapid adaptation to environmental stresses, detoxification of exogenous toxic compounds [36], and regulation of genes expression involved in metabolic processes [37].

We performed an RNAseq experiment to analyze the expression of *R. solani* AG1-IA genes during the pathogenesis of susceptible (TN1 and BPT5204) and tolerant (Tetep and Pankaj) rice genotypes. Genes expressed in a cultivar specific manner, as well as commonly expressed genes, were analyzed to understand the general and specific interaction of *R. solani* with different rice genotypes. Genes encoding key proteins such as secreted proteins, cytochrome P450, and transcription factors were utilized for further analysis. Later, the study was focused on genes encoding CWDE, specifically the pectin degradation enzymes. A comprehensive expression profile of genes encoding pectin degradation enzymes of *R. solani* AG1-IA was developed by quantitative real-time PCR analysis at different time points of infection, rice genotypes, tissues, and growth stages. Also, we analyzed the expression of these genes from mild and severe strains of *R. solani* AG1-IA.

## 2. Materials and Methods 

### 2.1. Plant Material, Pathogen Strains, and Sample Preparation 

**Figure 1:**
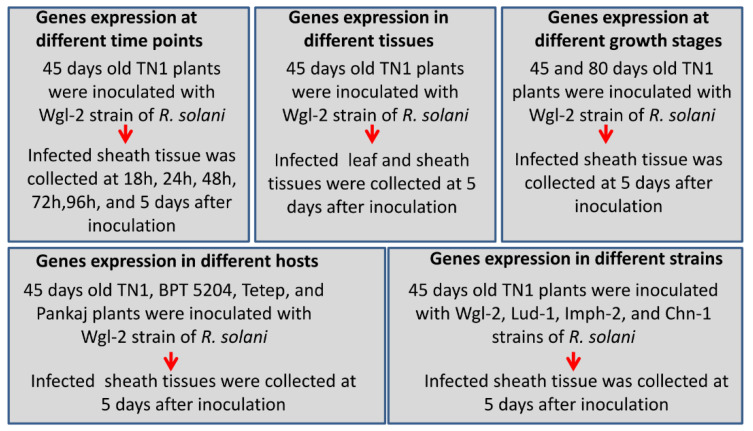
Details of sample preparation for the qRT-PCR experiments. The samples were prepared to evaluate the fungal genes expression at different time points of infection and in different tissues, growth stages, and genotypes of rice. Gene expression was also analyzed in different strains of *R. solani*.

*Oryza sativa* ssp. *indica* cultivars TN1, BPT5204 (Samba Mahsuri), Tetep, and Pankaj were used in this study. Among these, TN1 and BPT5204 are highly susceptible while Tetep and Pankaj are moderately tolerant against sheath blight disease [16,38,39]. The *R. solani* AG1-IA strains Wgl-2, Lud-1, Imph-2, and Chn-1 were used for plant inoculation. Among these, Wgl-2 and Lud-1 are highly virulent whereas Imph-2 and Chn-1 are moderately virulent [40]. Fungal sclerotia were cultured on PDA (39 g/L; Potato Dextrose Agar; HiMedia, Mumbai, India) medium at 28 °C. The freshly grown equal-sized sclerotia were used for plant inoculation by the methods described in our previous reports [16,41]. The sclerotia were placed on plants kept at warm and moist conditions. For analyzing the time dependent regulation of fungal genes expression, sheath tissue of TN1 plants was harvested after 18 h, 24 h, 48 h, 72 h, 96 h, and 5 days of inoculation with *R. solani* AG1-IA strain Wgl-2. To analyze the genes expression in different tissues, leaf and sheath of TN1 were harvested after 5 days of fungal inoculation. Infected sheath tissue of TN1 was harvested from 45 days (vegetative stage) and 80 days (reproductive stage) old plants to investigate the fungal genes expression at different growth stages of host plant. Samples from different rice cultivars (TN1, BPT5204, Tetep, and Pankaj) and rice plant (TN1) inoculated with different strains of fungi were also harvested to analyze the expression of fungal genes regulated by different rice genotypes and *R. solani* strains (Figure 1). 

### 2.2. RNAseq and Sequence Analysis

Leaf and sheath tissues of 45 days old rice plants (TN1, BPT5204, Tetep, and Pankaj) at vegetative stage were inoculated with Wgl-2 strain of *R. solani.* The infected rice tissues (sheath and leaf) collected at 5 days post-inoculation were used for RNA isolation and sequencing on the HiSeq 2500 system (Nucleome Informatics Pvt. Ltd., Hyderabad, India). Samples from three biological replicates (3 plants per replicate) were pooled for RNA sequencing to get maximum coverage of expressed transcripts of fungi. In our previous report [41], we used this RNAseq data to report the expression profile of only 16 pectin degrading genes of *R. solani* expressed in different rice cultivars. Whereas in this study, the whole genome transcriptome data of *R. solani* expressed in four rice cultivars were analyzed in detail. The data were used to study the gene expression pattern of secreted proteins, cytochrome P450 monooxygenases, transcription factors, and carbohydrate-active enzymes. The detailed procedure of sample preparation, RNA isolation, quality assessment, pair-end (2 × 100 bp) cDNA sequencing libraries preparation, sequencing, and functional annotation of transcripts was described in our previous study [41]. Briefly, the transcripts of the pure culture of Wgl-2 were used to filter the fungal transcripts from sequencing data of infected plant tissue. The featureCounts method of Subread software was used to analyze the gene expression in this experiment (http://subread.sourceforge.net/). FPKM (fragments per kilobase per million mapped reads) value of 1 was set as a threshold to determine if the gene is expressed or not. Gene annotation and gene ontology analysis of fungal transcripts were done as reported earlier [41]. 

### 2.3. Quantitative Real-Time PCR (qRT-PCR)

Thirty genes of *R. solani* encoding pectin degradation enzymes (PDE) were identified from RNAseq. The expression profile of these genes was analyzed through qRT-PCR. An experiment was planned to quantify the expression of PDE genes under different treatments (Figure 1). Total RNA was isolated using the RNeasy Plant Mini Kit (Qiagen, Hilden, Germany). DNase treated RNA was normalized and used for cDNA synthesis using Prime Script RT Reagent Kit (TaKaRa Bio Inc., Kusatsu, Japan). The cDNA was treated with RNase. qRT-PCR primers were designed using the online software of Integrated DNA Technologies (Table S1). The qRT-PCR reaction was set using the SYBR premix Ex-Taq kit (TaKaRa, Japan) as per the manufacturer’s protocol and performed in PCR LC-96-well plates (Roche LightCycler 96; Roche). The 18S *R. solani* ribosomal DNA specific primers were used as an internal control [14]. The relative gene expression was analyzed by the ΔΔCt method and fold change was calculated by 2^-ΔΔCt^ [42]. Three biological replicates and three technical replicates were used for the experiment. The expression level of these genes in *R. solani* grown in PDA medium was used as a reference point for comparison. 

### 2.4. Statistical Analysis

The statistical analyses were performed using one-way analysis of variance (ANOVA) and Tukey test using GraphPad prism program version 5.0 (GraphPad Software, San Diego, USA). The *p*-Value of less than 0.05 was considered as statistically significant.

## 3. Results

### 3.1. RNA Sequencing and Sequence Statistics

**Figure 2:**
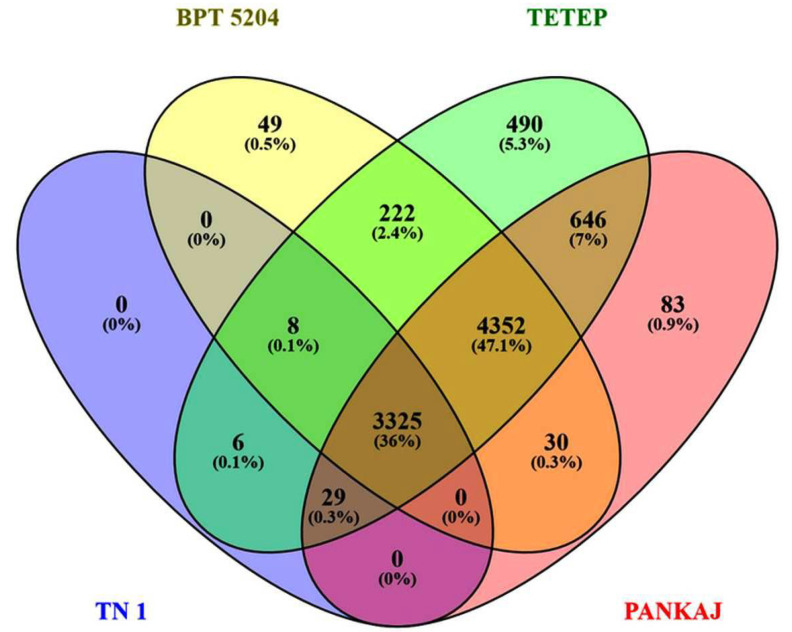
Venn diagram showing common and exclusive genes of *R. solani* expressed in TN1, BPT5204, Tetep, and Pankaj.

More than 121 million raw reads and 113 million clean reads were obtained from RNA sequencing of *R. solani* infected tissue of TN1, BPT5204, Tetep, and Pankaj. These reads were mapped to the rice genome and the filtered reads were mapped to the *R. solani* genome [43,44] and transcriptome [41]. More than 3.0 million reads of Tetep, 2.0 million reads of Pankaj, and 1.0 million reads of BPT5204 could be mapped to *R. solani* genome. However, in the case of TN1, only 36,904 reads were mapped which might be due to the rotting of tissue. TN1 is most susceptible to *R. solani* and was used as a susceptible check variety. These reads were utilized for assembly into contigs and gene identification. Details of the identified genes in each sample are given in Table S2. The sequence statistics are given in Table 1. Among the expressed genes of *R. solani*, 3325 genes were commonly expressed in all rice genotypes, while 49 (BPT5204), 490 (Tetep), and 83 (Pankaj) genes were specific to the genotypes, respectively (Figure 2). Among the genes expressed in a cultivar specific manner, a NUDIX domain-containing protein (AG1IA_02392) in BPT5204; NACHT domain-containing protein (AG1IA_06487) and BTB domain-containing protein (AG1IA_03906) in Pankaj; and sugar transporters (AG1IA_06806, AG1IA_02307), G-patch domain-containing protein (AG1IA_08655), OB_NTP_bind domain-containing protein (AG1IA_10057), ion transport protein (AG1IA_05437), ABC transporter (AG1IA_02225), cytochrome p450 (AG1IA_08418, AG1IA_09583, AG1IA_00743, and AG1IA_06232), response regulator receiver domain (AG1IA_08721), PHD domain-containing protein (AG1IA_03252), ubiquitin-conjugating enzyme (AG1IA_06203), and argonaute-like protein (AG1IA_04679) in Tetep; were identified (Table S3). Also, the majority of cultivars specific *R. solani* genes were annotated as hypothetical proteins.

### 3.2. Classification of Genes Based on Their Functions

The assembled transcripts were annotated for biological functions and further analysis was restricted to genes associated with essential roles of fungal growth and metabolism. 

#### Secreted Proteins and Cytochrome P450 Monooxygenases

Two hundred genes of *R. solani* encoding potentially secreted proteins showed expression during the infection of four rice genotypes. The expression pattern of these genes varied significantly among the four genotypes (Figure S1); however, we did not notice any distinguishable expression pattern of genes between the susceptible and tolerant rice genotypes. The highly expressed genes among all the genotypes were AG1IA_02674, AG1IA_03037, AG1IA_03226, AG1IA_05531, AG1IA_05601, AG1IA_06410, AG1IA_06970, AG1IA_07280, AG1IA_08493, AG1IA_09198, and AG1IA_10043. The genes such as AG1IA_00737, AG1IA_01858, AG1IA_03628, AG1IA_04547, AG1IA_05365, AG1IA_06115, AG1IA_07692, AG1IA_07735, AG1IA_08487, AG1IA_08781, AG1IA_08868, and AG1IA_09597 showed preferential expression in Tetep and Pankaj. Similarly, 58 putative cytochrome P450 (CYP) monooxygenases showed differential expression among the four rice genotypes (Figure S2); however, their expression level did not show a certain pattern among the susceptible and tolerant genotypes. AG1IA_03025, AG1IA_03629, and AG1IA_09807 showed high expression in all the genotypes while AG1IA_00013 and AG1IA_00318 expression was more in tolerant genotypes. 

### 3.3. Transcription Factors 

**Figure 3:**
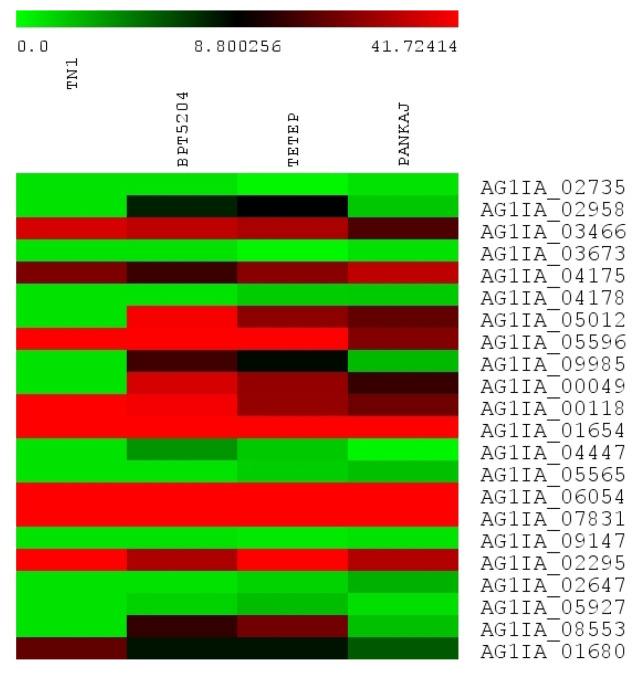
Heat map showing the expression of *R. solani* genes encoding transcription factors. The expression of transcription factor (TF) genes was analyzed in four rice cultivars. The values shown in bars at the top indicate FPKM of TF genes in different rice cultivars.

Twenty-two fungal genes encoding transcription factors (TFs) showed expression during the infection of rice cultivars. The identified TFs represented three categories: 1- basic-leucine zipper (bZIP) domain-containing TFs, 2- helix-loop-helix (HLH) TFs, and 3- zinc finger (Zn) TFs. The majority of the TFs belong to bZIP (9) followed by HLH (8), and Zn (5) (Figure 3). The bZIP TFs AG1IA_03466, AG1IA_04175, and AG1IA_05596; the HLH TFs AG1IA_00118, AG1IA_01654, AG1IA_06054, and AG1IA_07831; and the Zn TF AG1IA_02295 showed high expression in all the genotypes; however, a clear difference of expression pattern was not observed between susceptible and tolerant genotypes.

### 3.4. Carbohydrate Active Enzymes

**Figure 4:**
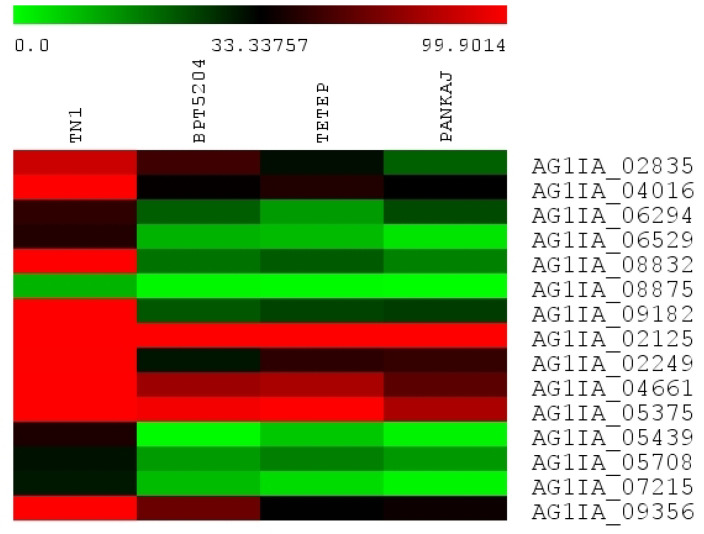
Heat map showing the expression of selected *R. solani* genes encoding carbohydrate-active enzymes. These genes showed very high expression in the most susceptible rice cultivar TN1. The values shown in bars at the top indicate FPKM.

Expression of 125 *R. solani* genes encoding carbohydrate-active enzymes (CAZymes) was recorded in four rice genotypes. The genes belonging to various categories such as glycoside hydrolases (GHs), glycosyl transferases (GTs), polysaccharide lyases (PLs), carbohydrate esterases (CEs), and non-catalytic carbohydrate-binding modules (CBMs) were identified (Figure S3). The highly expressed genes in all the four genotypes were AG1IA_03106, AG1IA_03699, AG1IA_04014, AG1IA_04569, AG1IA_05302, AG1IA_05475, AG1IA_06302, AG1IA_07787, AG1IA_09735, AG1IA_04084, AG1IA_00422, AG1IA_00523, AG1IA_06917, and AG1IA_08712. The genes AG1IA_04992, AG1IA_06708, AG1IA_07901, AG1IA_04214, AG1IA_07625, and AG1IA_04992 showed preferential expression in tolerant genotypes. AG1IA_05653 and AG1IA_06890 showed high expression in all the genotypes except TN1 where no expression was recorded. Interestingly, AG1IA_02835, AG1IA_04016, AG1IA_06294, AG1IA_06529, AG1IA_08832, AG1IA_08875, AG1IA_09182, AG1IA_02125, AG1IA_02249, AG1IA_04661, AG1IA_05375, AG1IA_05439, AG1IA_05708, AG1IA_07215, and AG1IA_09356 encoding GHs, GTs, and PLs showed very high expression in TN1 as compared to other genotypes (Figure 4).

### 3.5. Pectin Degrading (PD) Enzymes

Sixteen genes (AG1IA_09779, AG1IA_07743, AG1IA_07436, AG1IA_07435, AG1IA_06212, AG1IA_02891, AG1IA_02890, and AG1IA_02889 encoding pectinesterases, and AG1IA_02200, AG1IA_06500, AG1IA_00634, AG1IA_01811, AG1IA_02234, AG1IA_03368, AG1IA_04727, and AG1IA_09419 encoding polygalacturonases) showed expression in the RNAseq data obtained from four rice genotypes. In our previous study [41], we showed that a gene involved in pectin degradation is an important pathogenicity factor. Knockdown of AG1IA_04727 encoding polygalacturonase showed a drastic reduction in damage and symptoms of sheath blight disease. Considering the important role of PD genes in pathogenesis and plant cell wall degradation, the experiments were conducted for a thorough analysis of the expression profile of these genes. The expression of these genes was quantified by qRT-PCR under different treatments as described below. To make it easy to understand, the gene symbol AG1IA has been replaced by PE (for pectinesterase coding genes) or PG (for polygalacturonase coding genes) in sections described below.

#### 3.5.1. Expression Analysis of PD Genes at Different Time Points after Infection

In our previous study [41], expression analysis of AG1IA_04727 encoding a polygalacturonase suggested its differential regulation at different time points of infection. Therefore, a comprehensive analysis of PD genes expression was performed at different time points of pathogen infection. In addition to the 16 PD genes mentioned above, the expression of 14 other genes involved in pectin degradation was analyzed at different time points of infection. These genes encode polysaccharide lyase family 1 protein (AG1IA_04740, AG1IA_06890, AG1IA_09286, and AG1IA_09356), polysaccharide lyase family 3 protein (AG1IA_05447), polysaccharide lyase family 4 protein (AG1IA_06377), rhamnogalacturonase B (AG1IA_01129, AG1IA_06618, and AG1IA_06619), pectate lyase domain-containing protein (AG1IA_03046), pectate lyase B (AG1IA_00690), putative pectate lyase C (AG1IA_10120), and galactan 1,3-beta-galactosidase (AG1IA_06157, AG1IA_07341).

**Figure 5:**
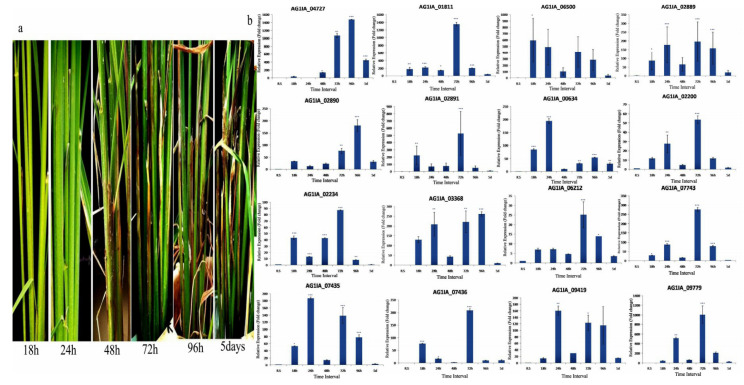
(**a**) Disease symptoms of *R. solani* Wgl-2 infection in susceptible rice cultivar TN1 after 18 h, 24 h, 48 h, 72 h, 96 h, and 5 days of inoculation. Clear visible sheath blight disease symptoms were observed only after 72 h of inoculation. (**b**) qRT-PCR expression analysis of 16 pectin degrading (PD) genes (polygalacturonases and pectinesterases) at different time points after fungal inoculation. X-axis: samples (R.s: *R. solani* cultured in potato dextrose agar medium, 18 h to 5 d: *R.solani* inoculated sheath tissue of TN1 harvested at different time points); Y-axis: Expression level of the individual PD genes relative to their expression in *R. solani* grown in PDA. Error bars indicate the mean ± S.E. of three biological replicates. Statistical significance was determined by performing one-way ANOVA analyses followed by a Tukey’s test (* = *p* < 0.01, ** = *p* < 0.001, and *** = *p* < 0.0001).

While comparing the expression of the transcripts of *R. solani* grown in PD medium with *R. solani* of infected rice tissue, all the 16 PD genes showed induced expression in infected rice tissue. After 18 h of fungal inoculation, maximum induction of PG_06500 (594 fold) followed by PE_02891 (221 fold), PG_01811 (180 fold), PG_03368 (129 fold), PG_00634 (82 fold), PE_07436 (74 fold), PE_02889 (70 fold), PE_07435 (52 fold), PE_09779 (48 fold), and PG_02234 (43 fold) was observed. After 24 h of inoculation, maximum induction of PE_09779 (517 fold) followed by PG_06500 (486 fold), PG_01811 (221 fold), PG_03368 (214 fold), PG_00634 (194 fold), PE_07435 (186 fold), PE_02889 (174 fold), PG_09419 (165 fold), and PE_07743 (89 fold) was observed. After 48 h of fungal inoculation, maximum induction of PG_01811 (151 fold), followed by PG_04727 (142 fold), PG_06500 (102 fold), PE_09779 (65 fold), PE_02889 (65 fold), PG_02234 (42 fold), and PG_03368 (41 fold) was observed. Interestingly, at this time point, we did not observe the induction of genes as high as in other time points. Also, the number of genes showing induction was less as compared to other time points. After 72 h of inoculation, maximum induction of PG_01811(1353 fold) followed by PG_04727 (1052 fold), PE_09779 (1000 fold), PE_02891 (526 fold), PG_06500 (233 fold), PE_07743 (280 fold), PG_03368 (206 fold), PE_07436 (203 fold), PE_02889 (190 fold), PG_09419 (137 fold), PE_07435 (121 fold), PG_02234 (87 fold), PE_02890 (74 fold), and PG_02200 (53 fold) was observed. This stage of infection seems to be most crucial, as the degree of induction, as well as the number of induced genes, was maximum at this time point. After 96 h of inoculation, maximum induction of PG_04727 (1478 fold) followed by PG_06500 (286 fold), PG_03368 (260 fold), PG_01811 (214 fold), PE_09779 (205 fold), PE_02890 (176 fold), PG_09419 (171 fold), PE_02889 (158 fold), PE_07743 (80 fold), PE_07435 (75 fold), and PG_00634 (53 fold) was observed. After 5 days of inoculation, the maximum induction of PG_04727 (444 fold) followed by PG_01811 (43 fold) and PG_06500 (41 fold) was observed. Among all the genes, PG_04727 was highly induced from 48 h to 5 days, PG_01811 was highly induced from 18 h to 5 days, and PE_09779 was highly induced from 24 h to 96 h. PE_06212 did not show considerable induction at any time point (Figure 5). In general, polygalacturonase encoding genes showed relatively higher expression than pectinesterase coding genes, specifically at later time points of infection.

**Figure 6:**
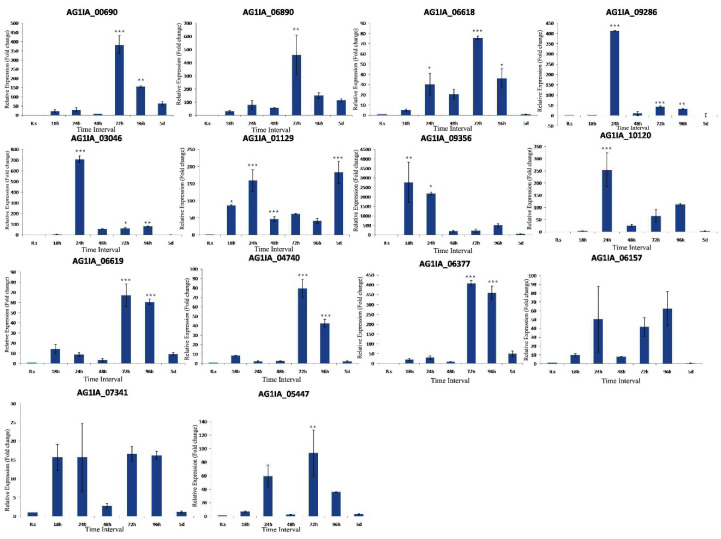
qRT-PCR expression analysis of 14 PD genes at different time points after fungal inoculation. X-axis: samples (R.s: *R. solani* cultured in potato dextrose agar medium, 18 h to 5 d: *R. solani* inoculated sheath tissue of TN1 harvested at different time points); Y-axis: Expression level of the individual PD genes relative to their expression in *R. solani* grown in PDA. Error bars indicate the mean ± S.E. of three biological replicates. Statistical significance was determined by performing one-way ANOVA analyses followed by a Tukey’s test (* = *p* < 0.01, ** = *p* < 0.001, and *** = *p* < 0.0001).

Among the other 14 genes (Table S4) involved in pectin degradation, differential regulation of genes at the different time course of infection was noticed. AG1IA_01129 and AG1IA_09356 showed 86 and 2762 fold induction after 18 h of inoculation, respectively. Six genes (AG1IA_06618-30 fold, AG1IA_09286-412 fold, AG1IA_03046-707 fold, AG1IA_01129-158 fold, AG1IA_09356-2187 fold, and AG1IA_10120-254 fold) showed significant induction after 24 h of inoculation. At the 48 h inoculation time point, we did not notice significant induction of most of the genes. Nine genes (AG1IA_00690-381 fold, AG1IA_06890-458 fold, AG1IA_06618-76 fold, AG1IA_06619-67 fold, AG1IA_03046-62 fold, AG1IA_04740-79 fold, AG1IA_06377-407 fold, AG1IA_09286-43 fold, and AG1IA_05447-93 fold) showed significant induced expression after 72 h of inoculation. Seven genes (AG1IA_00690-155 fold, AG1IA_06618-36 fold, AG1IA_09286-32 fold, AG1IA_03046-79 fold, AG1IA_06619-61 fold, AG1IA_04740-43 fold, and AG1IA_06377-358 fold) showed significant induction after 96 h of inoculation. AG1IA_01129 showed 182 fold induced expression at five days of inoculation. Similar to PG and PE genes, these PD genes also showed maximum activity at the 72 h inoculation time point and least activity at 48 h of inoculation (Figure 6).

#### 3.5.2. Expression Analysis of PD Genes in Different Rice Genotypes

**Figure 7:**
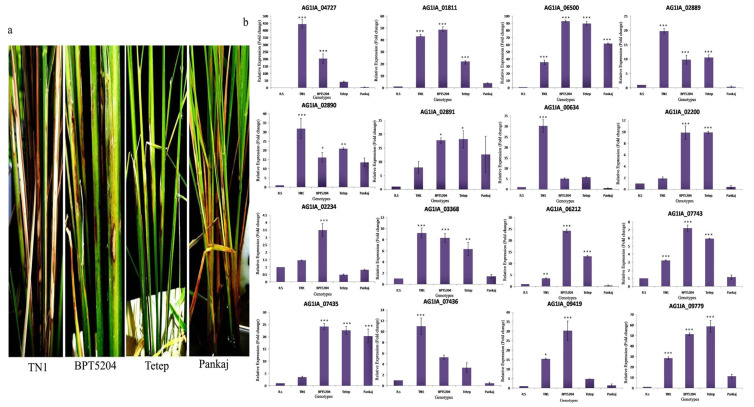
(**a**) The sheath blight disease symptoms on TN1, BPT5204, Tetep, and Pankaj upon inoculation of *R. solani* Wgl-2. (**b**) qRT-PCR expression analysis of 16 PD genes (polygalacturonases and pectinesterases) in different rice genotypes after *R. solani* inoculation. X-axis: samples (*R.solani* inoculated sheath tissue of four genotypes); Y-axis: Expression level of the individual PD genes relative to their expression in *R. solani* grown in PDA. Error bars indicate the mean ± S.E. of three biological replicates. Statistical significance was determined by performing one-way ANOVA analyses followed by a Tukey’s test (* = *p* < 0.01, ** = *p* < 0.001, and *** = *p* < 0.0001).

PG_04727 showed maximum induction in TN1 (444 fold) followed by BPT5204 (203 fold). In the tolerant genotypes, Tetep and Pankaj, this gene showed 49.0 and 6.0 fold induction, respectively. PG_01811 showed 43.0, 49.0, 22.0, and 4.0 fold induction in TN1, BPT5204, Tetep, and Pankaj, respectively. PG_09419 showed 16.0, 30.0, 4.0, and 2.0 fold induction in TN1, BPT 5204, Tetep, and Pankaj, respectively. Other genes also showed induction during infection, although the expression pattern was not distinct between susceptible and tolerant genotypes. PG_06500, PE_02890, and PE_09779 showed high level of induction in all the genotypes. Out of the 16 PD genes, 12 genes showed lesser induction in Pankaj when compared with the other three genotypes (Figure 7).

#### 3.5.3. Expression Analysis of PD Genes in Leaf Blade and Sheath Tissues

**Figure 8:**
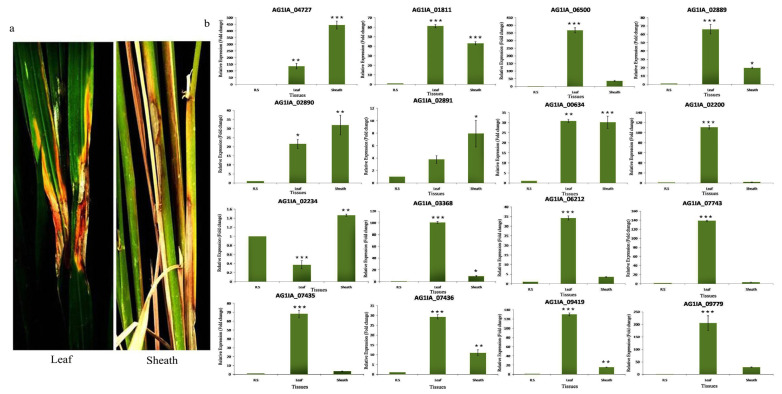
(**a**) The sheath blight disease symptoms on the leaf blade and sheath tissues of TN1 upon inoculation of *R. solani* Wgl-2. (**b**) qRT-PCR expression analysis of 16 PD genes (polygalacturonases and pectinesterases) in leaf blade and sheath tissues after *R. solani* inoculation. X-axis: samples (*R. solani* inoculated leaf blade and sheath tissues of TN1); Y-axis: Expression level of the individual PD genes relative to their expression in *R. solani* grown in PDA. Error bars indicate the mean ± S.E. of three biological replicates. Statistical significance was determined by performing one-way ANOVA analyses followed by a Tukey’s test (* = *p* < 0.01, ** = *p* < 0.001, and *** = *p* < 0.0001).

While comparing the expression level of 16 PD genes in the leaf blade and sheath tissues, we observed that the majority of the genes showed more induction in the leaf blade as compared to the sheath. PG_01811, PG_06500, PE_02889, PG_00634, PG_02200, PG_03368, PE_06212, PE_07743, PE_07435, PE_07436, PG_09419, and PE_09779 showed 61.0, 366.0, 67.0, 32.0, 116.0, 103.0, 34.0, 138.0, 68.0, 29.0, 135.0, and 205.0 fold enhanced expressions in leaf blade tissue, respectively, which was relatively very high when compared with the expression level of sheath tissue. PG_04727 and PE_02890 showed higher expression in sheath tissue (Figure 8).

#### 3.5.4. Expression Analysis of PD Genes in Different Growth Stages of Rice

**Figure 9:**
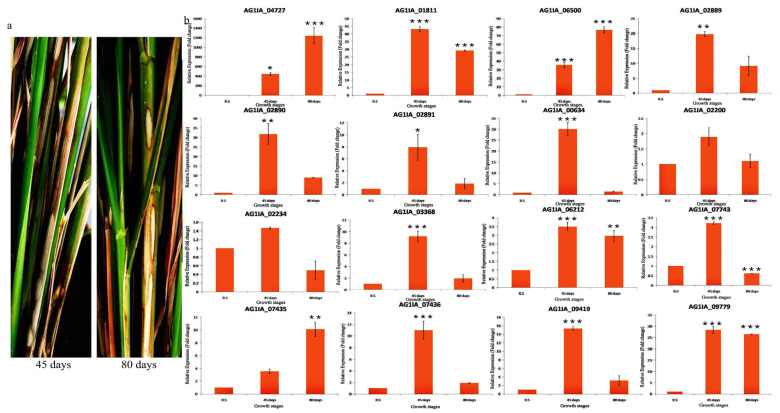
(**a**) The sheath blight disease symptoms on 45 days and 80 days old TN1 plants upon inoculation of *R. solani* Wgl-2. (**b**) qRT-PCR expression analysis of 16 PD genes (polygalacturonases and pectinesterases) in sheath tissue of 45 days and 80 days old TN1 plants after *R. solani* inoculation. X-axis: samples; Y-axis: Expression level of the individual PD genes relative to their expression in *R. solani* grown in PDA. Error bars indicate the mean ± S.E. of three biological replicates. Statistical significance was determined by performing one-way ANOVA analyses followed by a Tukey’s test (* = *p* < 0.01, ** = *p* < 0.001, and *** = *p* < 0.0001).

After infection, almost all the PD genes showed induced expression at both the growth stages of rice. While comparing both the growth stages, the majority of genes showed more induction at 45 days old-growth stage. Genes showing more induction at 80 days old-growth stage were PG_04727, PG_06500, and PE_07435. PE_09779 showed a similar level of induction at both the growth stages. PG_04727 showed maximum induction, i.e., 444 fold at 45 days and 1296 fold at 80 days growth stage (Figure 9). 

#### 3.5.5. Expression Analysis of PD Genes in Different Strains of *R. solani*

**Figure 10:**
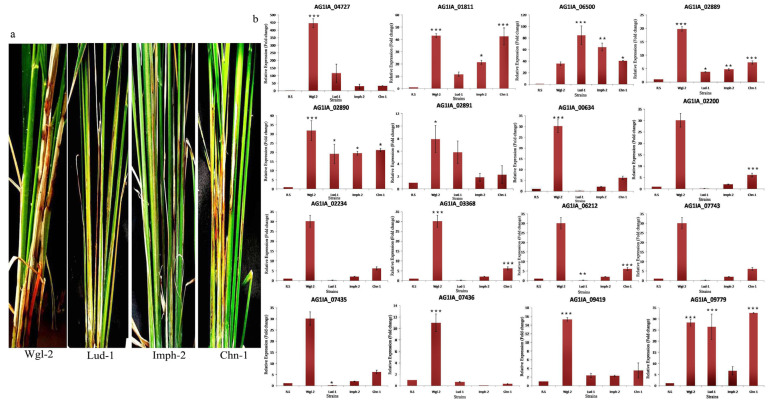
(**a**) The sheath blight disease symptoms on TN1 plants upon inoculation of different strains of *R. solani*. (**b**) qRT-PCR expression analysis of 16 PD genes (polygalacturonases and pectinesterases) in sheath tissue of TN1 after inoculation with different strains of *R. solani*. X-axis: samples (sheath tissue of TN1 inoculated with four different strains of *R. solani*); Y-axis: Expression level of the individual PD genes relative to their expression in *R. solani* grown in PDA. Error bars indicate the mean ± S.E. of three biological replicates. Statistical significance was determined by performing one-way ANOVA analyses followed by a Tukey’s test (* = *p* < 0.01, ** = *p* < 0.001, and *** = *p* < 0.0001).

PG_04727 was induced to the extent of 444 and 115 fold in highly virulent strains Wgl-2 and Lud-1, respectively. The milder strains Imph-2 and Chn-1 showed 28 and 34 fold induction of this gene, respectively. Other genes also showed induced expression in *R. solani* strains, but we did not notice any distinct expression pattern between highly virulent and mild strains. Among all the strains, Wgl-2 showed higher induction of the maximum number of genes. Notably, significant variation in the expression level of these genes was recorded among different strains (Figure 10).

## 4. Discussion

In a recent study, the host selection fitness mechanism was revealed by analyzing the expression of *R. solani* AG1IA genes during the infection of rice, soybean, and corn [26]. Considering the enormous genetic variations within the rice crop, we performed an RNAseq experiment using two tolerant and two susceptible rice genotypes to analyze the expression of *R. solani* genes during infection. Notably, 49, 83, and 490 *R. solani* genes showed preferential expression in BPT5204, Pankaj, and Tetep, respectively. This suggests that regulation of *R. solani* genes during the infection is significantly influenced by the genetic makeup of rice cultivars. In the susceptible cultivar BPT5204, the *R. solani* showed preferential expression of a nucleoside diphosphate-linked moiety X (Nudix) domain-containing protein (AG1IA_02392). Nudix effectors are secreted proteins that play a crucial role in pathogenesis [45]. Over-expression of CtNUDIX in *Magnaporthe oryzae* resulted in incompatibility with the barley (host) [46]. Nudix effectors have been characterized in very few pathosystems; therefore, it would be important to decipher the role of AG1IA_02392 in *R. solani* AG1 IA pathogenesis. NACHT domain-containing protein (AG1IA_06487) and BTB domain-containing protein (AG1IA_03906) showed preferential expression in Pankaj. The critical role of NACHT domain proteins has been suggested during mycorrhizal symbiosis [47]. POB1, a BTB domain E3 ligase protein of plants, suppresses hypersensitive response programmed cell death (HR-PCD), which is a well-known plant immunity mechanism to restrict pathogen and disease development [48,49]. Since Pankaj is a tolerant genotype [16], *R. solani* may encode proteins to suppress HR-PCD mediated host immunity. The functional characterization of pathogen-derived BTB domain-containing protein AG1IA_03906 may shed light on the counter-defense mechanism of *R. solani*. The fungal pathogen also showed preferential expression of several genes in Tetep. These genes encode proteins such as sugar transporters, ion transport protein, PHD domain-containing protein, cytochrome P450, argonaute-like protein, and ubiquitin-conjugating enzyme that might play a critical role in the pathogenesis of *R. solani*. Some of these candidate genes can be high priority targets for researchers working in the area of fungal functional genomics. Notably, the majority of cultivars (host) specific *R. solani* genes were annotated as hypothetical proteins that emphasize the necessity for functional characterization of fungal genes to reveal the processes of disease development. 

We also surveyed the expression of secreted protein genes, CYP450s, and transcription factors in four genotypes. The highly expressed among all the genotypes and preferentially expressed genes in tolerant genotypes were identified. Differential expression of these genes in rice genotypes suggests their important role in host–pathogen interaction. CYP450s play diverse roles in fungal cellular processes such as secondary metabolic pathways and detoxification of chemicals/pesticides, which allow fungi to grow in hostile surroundings [50]. These are key target genes/proteins for the development of fungicide molecules. Similarly, fungal TFs have become a central subject of study in recent times due to their crucial role in regulating gene expression and functions, and evolution [51]. The identified TF genes in this study need to be characterized in detail for establishing their roles in fungal metabolism and disease development. Further, we analyzed the expression of fungal encoded carbohydrate-active enzymes (CAZymes) in four genotypes. Genes such as AG1IA_03106, AG1IA_03699, AG1IA_04014, AG1IA_04569, AG1IA_05302, AG1IA_05475, AG1IA_06302, AG1IA_07787, AG1IA_09735, AG1IA_04084, AG1IA_00422, AG1IA_00523, AG1IA_06917, and AG1IA_08712 showed high expression in all the genotypes. CAZymes play a critical role in the degradation of the plant cell wall, a principal barrier for pathogens or saprophytes to invade. These genes also determine the growth efficiency and aggressiveness of phytopathogens [52,53]. Interestingly, a large number of genes encoding GHs, GTs, and PLs showed very high expression in the extremely susceptible cultivar TN1. This could explain the maximum rotting of infected tissue in TN1. It should be noted that we could map the minimum number of sequence reads from TN1 due to rotten tissue.

Pectin is one of the most important and complex components of plant cell wall [54]. It is also a vital contributor to plant immunity [55] as demonstrated successfully through mutations of genes associated with pectin biosynthesis [56]. Degradation of pectin by fungal encoded pectinases or pectin degrading enzymes helps in loosening the plant cell wall for penetration and colonization of fungus and serves as a carbon source for its growth and metabolism. Pectin degrading enzymes also function as virulence factors in phytopathogenic fungi [41,57,58]. These genes can also be the key targets for RNAi or genome editing based development of sheath blight disease resistance in rice [41]. However, very few studies have attempted to characterize the role of these genes in the infection process of *R. solani* and disease development. In this study, 30 PD genes were identified from RNAseq data of four rice genotypes. The qRT-PCR experiment was done to analyze the expression behaviour of these genes under various circumstances. 

Expression analysis of PD genes at different time points of infection suggested early and late responsive genes that might play specific roles during pathogenesis. Analysis indicates preferential recruitment of specific PD genes at different time points for the degradation of pectin. This could also be a strategy of fungus to degrade the diverse group of polysaccharides present in pectin, which comprises of 17 different monomers containing more than 20 different glycosidic linkages [59,60]. The maximum number of PD genes showed very high induction at 72 h after inoculation, suggesting this time point as the most crucial for disease establishment. Notably, sharp induction of PG_04727, PG_01811, PG_06500, PE_09779, and AG1IA_01129 was observed at more than one time point; hence, these are top priority candidate genes for detailed characterization to establish their role in the infection process. This study can help in providing the molecular basis of disease initiation and progression. PG_04727, PG_01811, and PG_09419 showed significantly higher induction in susceptible cultivars, TN1 and BPT52014, than in that of the tolerant cultivars, while PG_06500, PE_02890, and PE_09779 showed high expression in both susceptible and tolerant cultivars. A deeper understanding of the function of these genes would help in deciphering the susceptibility or tolerance mechanisms of rice against *R. solani*. Differential regulation of these genes in different rice cultivars suggests that the expression of these fungal genes is influenced by the genetic composition or phenotype of the host. This could also be due to the differences in the architecture of pectin of different rice genotypes. The composition and ratio of different polysaccharides, as well as their level of substitution of pectin, varies in different species and tissues of plants [61,62]. Interestingly, the majority of PD genes showed the least induction in Pankaj as compared to the other three genotypes. In our previous study, we showed the least damage in Pankaj by *R. solani*, which was supported by expression analysis of rice genes, microRNAs, and proteome analysis [16]. Pankaj has been utilized as a donor parent for sheath blight resistance trait to develop many rice varieties through the All India Coordinated Rice Improvement Project (AICRIP) trials [63]. 

The expression of most of the PD genes was high in the leaf blade as compared to sheath tissue. This could be due to higher pectin content in the leaf blade than the sheath [64]. Degradation of leaf blade cells would require increased activity or amount of fungal PD enzymes. This study partly explains the reason for the preference of sheath tissue as a primary point of infection by *R. solani*. Penetration of sheath tissue would require less energy and resources of fungus due to lesser pectin content in cell walls. Between the two stages of rice growth, induction of PD genes was more in 45 days old inoculated plants than in the 80 days old inoculated plants. The maximum tillering stage of rice is more appropriate for sheath blight disease development compared to booting or milking stages, which could be due to higher activity of fungal PD enzymes at the maximum tillering stage. We did not notice significant differences in expression of PD genes between the highly virulent and mild strains of *R. solani*, except for the PG_04727 which showed significantly enhanced expression in highly virulent strains (444 and 115 fold in Wgl-2 and Lud-1, respectively) than in the mild strains (28 and 34 fold in Imph-2 and Chn-1, respectively). In our previous study, we showed AG1IA_04727 to be one of the key pathogenicity determinants of *R. solani* [41]. This gene may also be a virulence factor. Significant variations in the expression level of PD genes among four *R. solani* strains suggest that these genes might have diverged and evolved with differential regulation capacity in different strains. 

## 5. Conclusions

The whole genome-transcriptome of *R. solani* AG1 IA generated by RNAseq from four fungal infected rice genotypes has helped better understand the pathogen genomics. The analysis of fungal genes involved in general and specific host–pathogen interactions revealed the priority genes for functional studies and a deeper understanding of pathogen biology. *R. solani* genes encoding secreted proteins, cytochrome P450, and transcription factors were all investigated in four rice genotypes to study the differential response of genes involved in essential functions of the pathogen. Expression of CWDE genes, particularly the genes involved in pectin degradation, was thoroughly analyzed for understanding the temporal and spatial regulation of fungal genes. The expression under various circumstances, such as at different time points of fungal infection, and also in different tissues, growth stages, and genotypes of the host plant, was analyzed. This should not only help in deciphering the molecular events associated with plant cell wall degradation during pathogenesis, but it also provides a clue to select the target genes for controlling the sheath blight disease by employing either RNAi or genome editing approaches. This study may even have utility for industries involved in the production of commercial pectinolytic enzyme mixtures for enzymatic hydrolysis of pectin.

## Figures and Tables

**Table 1:** Summary of *R. solani* transcriptome data obtained through RNA sequencing of fungal infected tissues of four rice cultivars. The sequences were filtered to acquire fungal encoded transcripts.

	TN1	BPT5204	Tetep	Pankaj
Raw Reads	31,796,190	22,300,547	34,215,148	33,660,702
Clean reads	29,709,930	20,923,575	31,231,404	31,272,956
Total mapped	36,904 (0.124214%)	1,126,634 (2.69226%)	3,751,721 (12.0127%)	2,081,471 (6.65582%)
Multiple mapped	5424 (0.0182565%)	63,940 (0.152794%)	78,081 (0.250008%)	57,214 (0.18295%)
Uniquely mapped	31,480 (0.105958%)	1,062,694 (2.53947%)	3,673,640 (11.7626%)	2,024,257 (6.47287%)
Read-1	16,224 (0.054608%)	552,367 (1.31996%)	1,895,574 (6.06945%)	1,049,157 (3.35484%)
Read-2	15,256 (0.0513498%)	510,327 (1.2195%)	1,778,066 (5.6932%)	975,100 (3.11803%)
Reads map to +	16,810 (0.0565804%)	533,654 (1.27525%)	1,854,599 (5.93825%)	1,024,606 (3.27633%)
Reads map to –	14,670 (0.0493774%)	529,040 (1.26422%)	1,819,041 (5.8244%)	999,651 (3.19654%)
Splice reads	9423 (0.0317167%)	405,652 (0.969366%)	1,634,976 (5.23504%)	1,005,575 (3.21548%)
Non-splice reads	22,057 (0.0742412%)	657,042 (1.5701%)	2,038,664 (6.52761%)	1,018,682 (3.25739%)

## Data Availability

The analyzed data are published in this article and are also provided as supplementary information. The raw data of RNAseq generated in this study have been deposited in Sequence Read Archive (SRA) database under BioProject ID PRJNA612594.

## Supplementary Materials

The following are available online at https://www.mdpi.com/2309-608X/6/2/71/s1, Figure S1: Heat map showing the expression of *R. solani* genes encoding secreted proteins. The expression of these genes was analyzed in four rice cultivars. The values shown in bars at the top indicate FPKM, Figure S2: Heat map showing the expression of *R. solani* genes encoding cytochrome P450s. The expression of these genes was analyzed in four rice cultivars. The values shown in bars at the top indicate FPKM, Figure S3: Heat map showing the expression of *R. solani* genes encoding carbohydrate-active enzymes. The expression of these genes was analyzed in four rice cultivars. The values shown in bars at the top indicate FPKM, Table S1: List of primers used in this study, Table S2: The details of genes expressed in four rice genotypes. The values indicate the FPKM, Table S3: The genes and their annotation showing cultivar specific expression, Table S4: Annotation of 14 genes involved in pectin degradation.
